# Evaluation of the early-phase [^18^F]AV45 PET as an optimal surrogate of [^18^F]FDG PET in ageing and Alzheimer’s clinical syndrome

**DOI:** 10.1016/j.nicl.2021.102750

**Published:** 2021-07-01

**Authors:** Matthieu Vanhoutte, Brigitte Landeau, Siya Sherif, Vincent de la Sayette, Sophie Dautricourt, Ahmed Abbas, Alain Manrique, Anne Chocat, Gaël Chételat

**Affiliations:** a Inserm UMR-S U1237, Caen-Normandie University, GIP Cyceron, Caen, France; b Inserm U1077, Caen-Normandie University, École Pratique des Hautes Études, Caen, France; c University Hospital, Neurology Department, Caen, France; d University Hospital, Nuclear Medicine Department, Caen, France

**Keywords:** Early-phase [^18^F]AV45 PET, [^18^F]FDG PET, Proxy, Neurodegeneration, Optimization, Aβ, β-amyloid, AD, Alzheimer’s disease, AD-d, dementia due to AD, Alz-CS, Alzheimer’s clinical syndrome patients, aMCI, amnestic mild cognitive impairment, BACC, Balanced accuracy, CBF, cerebral blood flow, CD, Critical difference, DK, Desikan-Killiany, eAV45, early-phase [^18^F]AV45 PET, eHC, Elderly healthy controls, FCSRT, free and cued selective reminding test, [^18^F]AV45, ^18^F-Florbetapir, [^18^F]FDG, [^18^F]fluorodeoxyglucose, GM, Gray matter, lAV45, late-phase [^18^F]AV45 PET, FWE, Familywise error, MMSE, Mini-mental state examination, MRI, Magnetic resonance imaging, PET, positron emission tomography, PVE, partial volume effect, SUVR, standardized uptake value ratio, ROIs, Regions of interest, SVM, Support vector machine, yHC, young healthy controls

## Abstract

Dual-phase [^18^F]AV45 positron emission tomography (PET) is highly promising in the assessment of neurodegenerative diseases, allowing to obtain information on both neurodegeneration (early-phase; eAV45) and amyloid deposition (late-phase; lAV45) which are highly complementary; yet eAV45 needs further evaluation. This study aims at validating eAV45 as an optimal proxy of [^18^F]FDG PET in a large mixed-population of healthy ageing and Alzheimer’s clinical syndrome participants (n = 191) who had [^18^F]FDG PET, eAV45 and lAV45 scans. We found early time frame 0–4 min to give maximal correlation with [^18^F]FDG PET and minimal correlation with lAV45. Moreover, maximal overlap of [^18^F]FDG PET versus eAV45 associations with clinical diagnosis and cognition was obtained with pons scaling. Across reference regions, classification performance between clinical subgroups was similar for both eAV45 and [^18^F]FDG PET. These findings highlight the optimal use of eAV45 to assess neurodegeneration as a validated proxy of [^18^F]FDG PET. On top of this purpose, this study showed that combined [^18^F]AV45 PET dual-biomarker even outperformed [^18^F]FDG PET or lAV45 alone.

## Highlights

• Exhaustive vertex-wise quantitative analyses to validate eAV45 as an optimal proxy for ^18^F-FDG PET.
• Optimal early time frame 0–4 min maximizes both within- and inter-subject correlations of eAV45 with ^18^F-FDG PET.
• Optimal early time frame 0–4 min minimizes both within- and inter-subject correlations of eAV45 with lAV45.
• Balanced accuracies of neurodegenerative pattern overlap of both ^18^F-FDG PET and eAV45 are maximal with pons scaling.
• Classification performance between clinical subgroups was similar for both eAV45 and [^18^F]FDG PET.

## 1 Introduction

The early and differential diagnosis of Alzheimer’s disease (AD) is still challenging (McKhann et al., 2011) and critically needs to be improved. As the field is moving toward a biological definition of AD (Jack et al., 2018), the role of biomarkers in diagnosis is becoming predominant e.g. with the β-amyloid (Aβ)/Tau/Neurodegeneration (A/T/N) scheme. In addition to detailed clinical and neuropsychological information, and CSF data when available, neuroimaging biomarkers are particularly meaningful and informative as they provide complementary information on the degree and topography of Aβ, tau and neurodegeneration (Chételat et al., 2020, Jack et al., 2018, Teipel et al., 2015).

In clinical setting, [^18^F]fluorodeoxyglucose ([^18^F]FDG) positron emission tomography (PET), measuring the reduction of cerebral metabolic rate of glucose caused by loss of synaptic activity, is an acknowledged biomarker for neurodegeneration in ageing and AD (Chételat et al., 2020). It provides functional information about disease stage and symptom severity (Landau et al., 2011, Mosconi et al., 2010) with an improved sensitivity compared to magnetic resonance imaging (MRI) (Laforce et al., 2018). Complementarily, [^18^F]Florbetapir ([^18^F]AV45) is recognized as an effective Aβ-specific radiotracer for use in PET imaging, and thus a reliable biomarker for Aβ in ageing and AD. It provides pathological information particularly useful for early diagnosis (Wong et al., 2010). There is widespread evidence that combining pathological and functional neuroimaging biomarkers would improve diagnostic accuracy and the specification of disease progression (Chételat et al., 2020, Ossenkoppele et al., 2013, Teipel et al., 2015). However, multiple scans are necessary to get both complementary information, namely [^18^F]AV45 PET for Aβ and [^18^F]FDG PET for neurodegeneration, which dramatically increases costs, radiation exposure and examination time for the patients.

Interestingly, due to the high lipophilic nature of the [^18^F]AV45 tracer, accumulating evidence indicates that early-phase [^18^F]AV45 PET (eAV45) reflects cerebral blood flow (CBF), which in turn is tightly coupled to cerebral glucose metabolism measured on [^18^F]FDG PET (Paulson et al., 2010). Previous studies have shown a strong correlation of eAV45 with [^18^F]FDG PET uptake in AD (Hsiao et al., 2012, Kuo et al., 2017, Lin et al., 2016), eAV45 appearing as a promising proxy for [^18^F]FDG PET. Dual-phase [^18^F]AV45 PET could thus allow to obtain both Aβ (pathological with late acquisition; lAV45) and neurodegeneration (functional with early acquisition; eAV45) information based on a unique [^18^F]AV45 PET injection, that may ultimately circumvent the need for an additional [^18^F]FDG PET scan in the AD work-up. However, further methodological development is critically needed towards evaluating the optimal early time frame and preprocessing of eAV45 to maximize its sensitivity in a large sample including both young to elderly cognitively unimpaired volunteers and patients with Alzheimer’s clinical syndrome (Jack et al., 2018). Furthermore, none of earlier study assessed the potential of the [^18^F]AV45 PET dual-biomarker for automatic AD diagnosis compared to standard biomarkers for Aβ and neurodegeneration, which would be of greatest interest in clinical routine. In this study, we will use CBF as measured by eAV45 to address these gaps in knowledge, by carrying out comprehensive and complementary vertex-wise quantitative analyses according to various criteria to validate eAV45 as an optimal proxy for [^18^F]FDG PET. These criteria will include: (i) within- and inter- subject correlations between both modalities; (ii) assessment of how each modality relates with clinical diagnosis and cognition, and quantification of the overlaps between both modalities; and finally (iii) supervised machine learning classification algorithm within robust cross-validation scheme to assess, at the individual level, the discriminatory power of [^18^F]AV45 PET as a dual-biomarker for AD diagnosis compared to the standard biomarkers for Aβ and neurodegeneration. These analyses will take into consideration the effects of the most widely used reference regions for both [^18^F]AV45 and [^18^F]FDG tracers, of early Aβ binding contamination and of partial volume effect (PVE).

## 2 Materials and methods

**Fig. 1:**
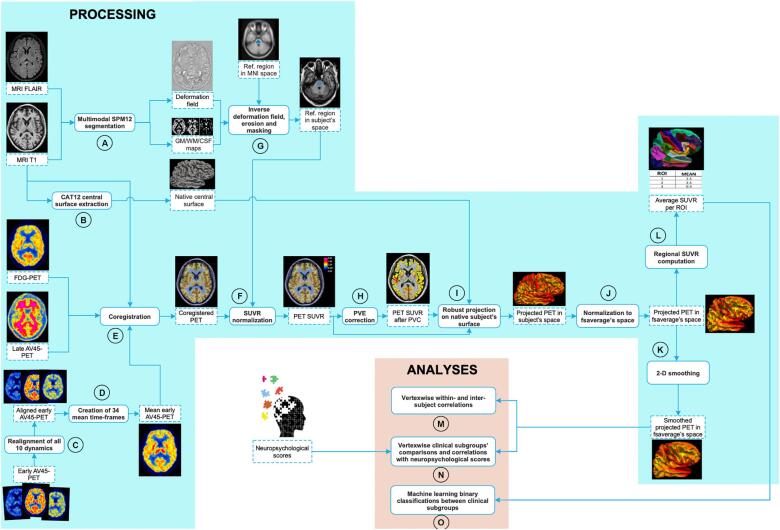
Flow chart for data processing and analyses.

The full processing and analysis sequences are shown by a flow chart in Fig. 1.

### 2.1 Study population

Sixty-one native French-speaking participants from the ‘Imagerie Multimodale de la Maladie d’Alzheimer à un stade Précoce’ (IMAP) Study (Caen, France) were included in the present study: 31 healthy controls including young (yHC; n = 16) and elderly (eHC; n = 15) volunteers with no cognitive impairment and 30 Alzheimer’s clinical syndrome patients (Alz-CS) of which 15 with amnestic mild cognitive impairment (aMCI) and 15 with dementia (AD-d). Inclusion and exclusion criteria of the IMAP Study are detailed in previous publications (Mutlu et al., 2017, Wirth et al., 2018). Patients were recruited from local memory clinics and selected according to corresponding internationally agreed criteria. Patients with aMCI were selected based on Petersen’s criteria for amnestic MCI (Petersen and Morris, 2005) and patients with AD-d fulfilled standard NINCDS-ADRDA clinical criteria for probable AD (McKhann et al., 1984). Further stratification by amyloid status was performed whenever needed (see section *Neuroimaging data processing*).

We also included 130 older adults who were cognitively unimpaired (eHC) from the Age-Well randomized controlled trial of the Medit-Ageing European Project (Poisnel et al., 2018), sponsored by the French National Institute of Health and Medical Research (INSERM). Those were recruited from the general population, older than 65 years, native French speakers, retired for at least 1 year, educated for at least 7 years, and able to perform within the normal range on standardized cognitive tests. The main exclusion criteria were safety concerns associated with magnetic resonance image (MRI) or PET scanning, evidence of a major neurological or psychiatric disorder (including alcohol or drug abuse), history of cerebrovascular disease, presence of a chronic disease or acute unstable illness, and current or recent medication usage that may interfere with cognitive functioning.

All 191 participants from both Projects included in the present study had, within a maximum period of 3 months, structural MRI, dual-phase [^18^F]AV45 PET and [^18^F]FDG PET scans, along with a neuropsychological examination (including the mini-mental state examination (MMSE) and the free and cued selective reminding test (FCSRT) (Grober and Buschke, 1987)). Participants’ demographics are displayed in Table 1.

**Table 1:** Demographics.

	yHC	eHC	aMCI	AD dementia	*P* value
Characteristics	(n = 16)	(n = 145)	(n = 15)	(n = 15)	
Female gender, n (%)	7 (43.8)	89 (61.4)	2 (13.3)	6 (40)	0.001
Age, years	40.2 ± 12.7	70.4 ± 4.6	75.6 ± 7.2*	69.4 ± 8.2	< 0.001
Education, years	12.6 ± 2.5	12.9 ± 3.1	12.3 ± 3.2	12.2 ± 4.3	0.79
MMSE	29 ± 1	29 ± 1 (40NA)	26 ± 3*	20 ± 5* (2NA)	< 0.001
Total Recall	15 ± 2 (9NA)	15 ± 1 (40NA)	10 ± 3*	5 ± 3* (5NA)	< 0.001
AV45 cortical SUVR [high-Aβ, n (%)]	1.16 ± 0.08 [2 (12.5)]	1.24 ± 0.16 [74 (51)]	1.48 ± 0.22* [13 (86.7)]	1.52 ± 0.26* [14 (93.3)]	< 0.001

*Aβ* amyloid β, *AD* Alzheimer’s disease, *aMCI* amnestic mild cognitive impairment, *eHC* elderly healthy controls, *yHC* young healthy controls, *MMSE m*ini mental state examination, *SUVR* standardized uptake value ratio.

Data presented as mean ± standard deviation. Categorical variables (gender, APOE ε4) were analyzed with Fisher’s exact test and continuous variables (age, education, MMSE, Total recall, SUVR) were analyzed with one-factor ANOVA with post-hoc Tukey’s HSD. The Aβ cutoff was determined using the specificity based cut off corresponding to the 95th percentile of the [^18^F]AV45 distribution in the neocortical mask of a group of 63 young healthy controls (age range = 20–40) (Jack et al., 2017).

*P < 0.05 versus eHC subjects.

The IMAP Study was approved by a regional ethics committee (Comité de Protection des Personnes Nord-Ouest III) and is registered with http://clinicaltrials.gov (number NCT01638949). The Age-Well randomized controlled trial was approved by the ethics committee (Comité de Protection des Personnes Nord-Ouest III, Caen, France; trial registration number: EudraCT: 2016–002441-36; IDRCB: 2016-A01767-44; ClinicalTrials.gov Identifier: NCT02977819). All participants gave written informed consent to the study prior to the investigation.

### 2.2 Neuroimaging data acquisition

All participants were scanned on the same MRI and PET cameras at the Cyceron Center (Caen, France): a 3 T Philips Achieva scanner (Philips Healthcare, Best, the Netherlands) and a Discovery RX VCT 64 PET-CT device (GE Healthcare, Milwaukee, WI, USA), respectively. The MRI sequences and parameters associated to the IMAP Study were described previously (Mutlu et al., 2017). For the Age-Well Study, high-resolution T1-weighted anatomical volume was first acquired using a 3D fast-field echo sequence (3D-T1-FFE sagittal; repetition time = 7.1 ms; echo time = 3.3 ms; flip angle = 6°; 180 slices with no gap; slice thickness = 1 mm; field of view = 256 × 256 mm^2^; in-plane resolution = 1 × 1 mm^2^). A high-resolution T2-weighted FLAIR anatomical volume was then acquired using a 3D inversion recovery sequence (3D-IR sagittal; repetition time = 4800 ms; echo time = 272 ms; inversion time = 1650 ms; flip angle = 40°; 180 slices with no gap; slice thickness = 1 mm; field of view = 250x250 mm^2^; in-plane resolution = 0.98x0.98 mm^2^).

Both FDG and dual-phase [^18^F]AV45 PET scans were acquired with a resolution of 3.76 × 3.76 × 4.9 mm^3^ (field of view = 157 mm). Forty-seven planes were obtained with a voxel size of 1.95 × 1.95 × 3.2 mm^3^. A transmission scan was performed for attenuation correction before the PET acquisition. For [^18^F]FDG PET, the participants were fasted for at least 6 h before scanning. After a 30 min resting period in a quiet and dark environment, 180 MBq of FDG was intravenously injected as a bolus. A 10 min PET acquisition scan began 50 min after injection. For [^18^F]AV45 PET, each participant underwent a 10-minutes early acquisition (composed of ten 1-minute dynamical frames) that began immediately after the intravenous injection of ~ 4 MBq/kg of [^18^F]AV45, and a 10-minutes late acquisition (beginning 50-minutes after injection).

### 2.3 Neuroimaging data processing

#### 2.3.1 Multimodal segmentation and central surface extraction

MRI data were segmented and normalized using the multimodal segment routine of the Statistical Parametric Mapping 12 (SPM12) software (Wellcome Trust Centre for Neuroimaging, London, UK), combining the information from different channels (T1w & FLAIR) in order to improve the segmentation accuracy (Fig. 1A). We applied an established algorithm implemented in the CAT12 toolbox (version r1450^1^) for simultaneously estimating cortical thickness and reconstructing the native central surfaces of the left and right hemispheres by using the projection-based thickness method (Dahnke et al., 2013) (Fig. 1B).

#### 2.3.2 eAV45 dynamics realignment and mean time-frames generation

The dynamic ten 1-minute images from eAV45 were initially corrected for motion using a 2-pass approach, first to the third frame and then to the 1 to 6 min mean (Hsiao et al., 2012) using SPM12 (Fig. 1C). We then iteratively generated the 34 possible combinations of eAV45 mean images corresponding to the sums of different frame ranges (eAV45[T_1_,T_2_], where T_1_ = 0, 1, 2 or 3 min and T_2_ = 1, 2, 3, 4, 5, 6, 7, 8, 9 or 10 min post-injection) (Fig. 1D).

#### 2.3.3 PET coregistration onto T1 MRI

All eAV45 mean images were coregistered onto their corresponding T1 MRI using the 1 to 6 min mean image as the reference (Hsiao et al., 2012) (Fig. 1E). In the same manner, static lAV45 and [^18^F]FDG PET were coregistered onto their corresponding T1 MRI (Fig. 1E).

#### 2.3.4 PET intensity normalization

To allow for inter-subject comparison, the PET images were then intensity normalized. Scaling was done in subject’s T1 native space by dividing the PET images by the mean uptake in selected reference regions, classically used for [^18^F]FDG PET and lAV45, derived from MNI space (see description below) (Fig. 1F). Thus, inverse deformation from MNI space to the subject’s T1, estimated above by the SPM12 normalization step, was applied to a reference region (Fig. 1G). An erosion by a 4-mm sphere and a masking by the corresponding thresholded tissue probability map (GM, WM or GM + WM; threshold = 0.5) were used to ensure that only appropriate voxels were considered when computing the mean uptake (Fig. 1G). lAV45 data were scaled using the cerebellar gray matter (GM), obtained from the Hammers atlas in MNI space (Hammers et al., 2003), as a reference region to obtain standardized uptake value ratio (SUVR) images (Fig. 1F). lAV45 uptake value was extracted in a predetermined neocortical mask (Besson et al., 2015, La Joie et al., 2013) (including the entire gray matter except the cerebellum, occipital and sensory motor cortices, hippocampi, amygdala, and basal nuclei). Then, this value was used to classify participant as amyloid positive or negative using a threshold of 1.22 [The threshold for positivity was determined on the basis of the mean lAV45 uptake values in the neocortical mask of a group of 63 young healthy controls (age range = 20–40), using the specificity based cut off corresponding to the 95th percentile of the lAV45 distribution since young healthy controls are likely to be relatively free of AD pathology (Jack et al., 2017)]. We considered seven different reference regions for scaling both eAV45 and [^18^F]FDG PET data in this study (Fig. 1F). These include: (i) cerebellum or cerebellar GM, obtained from the Hammers atlas in MNI space (Hammers et al., 2003), because of their low susceptibility to age-related or AD changes in metabolism (Herholz et al., 2002, M Bauer, 2013) and of the well preservation from Aβ plaques in these regions (Choi et al., 2012); (ii) global normalization, by using proportional scaling to a physiologically realistic reference value of 6.5 mg/100 mL/min as proposed in (Perani et al., 2014), which is widely employed in [^18^F]FDG PET research of neurodegenerative dementia (Yakushev et al., 2008); (iii) pons, obtained from the Pick atlas in MNI space (Maldjian et al., 2003), which seems to be metabolically least affected in AD (Minoshima et al., 1995) and more stable for lAV45 in MCI (Shokouhi et al., 2016a, Shokouhi et al., 2016b); (iv) a combination of both pons and cerebellum or pons and cerebellar GM, since composite regions may result in more accurate change measurements of lAV45 (Landau et al., 2015); and finally (v) cerebral WM regions (Gonneaud et al., 2017) because it seems to detect more stable and plausible longitudinal SUVR values of lAV45 (Fleisher et al., 2017).

#### 2.3.5 PET PVE correction

Finally, all scaled PET images were corrected for PVE using the 3-compartmental voxel-wise “Müller-Gärtner” method (Gonzalez-Escamilla et al., 2017, Muller-Gartner et al., 1992) (Fig. 1H).

#### 2.3.6 PET robust projection on native surface

Both PVE-non corrected and PVE-corrected PET images were then robustly projected on the subject’s cortical surface (Marcoux et al., 2018) (Fig. 1I). For each vertex, the projected PET signal was obtained by computing a weighted average of the PET signal intersecting the surfaces from 35% to 65% of the cortical thickness with a step t = 5% (i.e. the central surface corresponds to 50% of the cortical thickness). Using a normal distribution centered at the central surface, more weight was given to the surfaces located near this central surface as they have a higher probability to be well located within the cortex.

#### 2.3.7 Native surface PET normalization and 2D smoothing

Each native surface PET map was subsequently registered against the standard surface space (“fsaverage” template) (Fig. 1J) and smoothed using an 8 mm full width at half-maximum isotropic 2-dimensional Gaussian kernel (Fig. 1K).

#### 2.3.8 Extraction of mean SUVR from Desikan-Killiany parcellation

Lastly, mean values inside regions of interest (ROIs) from the Desikan-Killiany (DK) parcellation were extracted from all unsmoothed normalized PET surface maps (Fig. 1L).

#### 2.3.9 Semi-automatic quality check

A semi-automatic quality check of both reconstructed surfaces and smoothed normalized preprocessed PET surfaces was applied. Euler number and defect size were computed for each reconstructed surface to estimate the number and size of topology defects, while correlations between all participants’ preprocessed PET surfaces for each tracer were calculated to assess the homogeneity of our data sample. Visual inspection of native participant T1 MRI was executed for reconstructed cortical surfaces with high Euler number and/or defect size. In the same manner, visual inspection of native participant PET data was performed for preprocessed PET surfaces with global correlation lower than two standard deviations. Following these inspections, no participant had to be discarded from the statistical analysis.

### 2.4 Statistical analysis

#### 2.4.1 Demographical and clinical statistics

Between-group differences in demographic and clinical variables were assessed with one-factor ANOVAs (Group) with post-hoc Tukey’s HSD tests for continuous variables and χ^2^ tests for categorical variables in R (v.3.5.1).

All further statistical analyses on PET data were performed on both PVE-non corrected and PVE-corrected data.

#### 2.4.2 Vertexwise within- and inter-subject correlations

The vertex-wise within-subject Pearson’s correlation between smoothed normalized eAV45[T_1_,T_2_] and [^18^F]FDG PET maps, and eAV45[T_1_,T_2_] and lAV45 maps were calculated in R (v.3.5.1) for comparison among all early time frame ranges (Fig. 1M). To assess the statistical significance of differences among this large set of early time frames, we performed a non-parametric Friedman test (Hollander et al., 2013) comparing the within-subject correlation of the 34 different early time frames simultaneously across multiple participants, for each of the correlations separately. The significantly highest vertex-wise within-subject correlation between eAV45[T_1_,T_2_] and [^18^F]FDG PET maps were used to select the best early time frame ranges. Then, the vertex-wise inter-subject Pearson’s correlation maps between smoothed normalized eAV45[T_1_,T_2_] and [^18^F]FDG PET maps, and eAV45[T_1_,T_2_] and lAV45 maps were calculated for comparison among those best early time frame ranges, for each reference region used for scaling (Fig. 1M). In order to obtain these vertex-wise inter-subject correlation maps, we applied a permutation inference for generalized linear models (PALM, version alpha 115) to provide exact control for false positives while making only weak assumptions about the data (Winkler et al., 2014). The number of permutations was set to 10,000 and vertex-wise inter-subject correlation coefficients were computed from the Student’s t statistic as:

(1) $$r=sign\left(t\right)\sqrt{\frac{t^{2}}{N-rank\left(M\right)+t^{2}}}$$

where, r is the correlation coefficient; t is the Student’s t statistic; N is the number of observations (participants); and M is the matrix of explanatory variables.

Thus, the optimal early time frame of [^18^F]AV45 PET was determined, among the best within-subject early time frame ranges, as the one that both maximized the inter-subject correlation of eAV45[T_1_,T_2_] with [^18^F]FDG PET and minimized the inter-subject correlation of eAV45[T_1_,T_2_] with lAV45 whatever the type of scaling used.

#### 2.4.3 Vertexwise clinical subgroups’ comparisons and correlations with neuropsychological scores

**Fig. 2:**
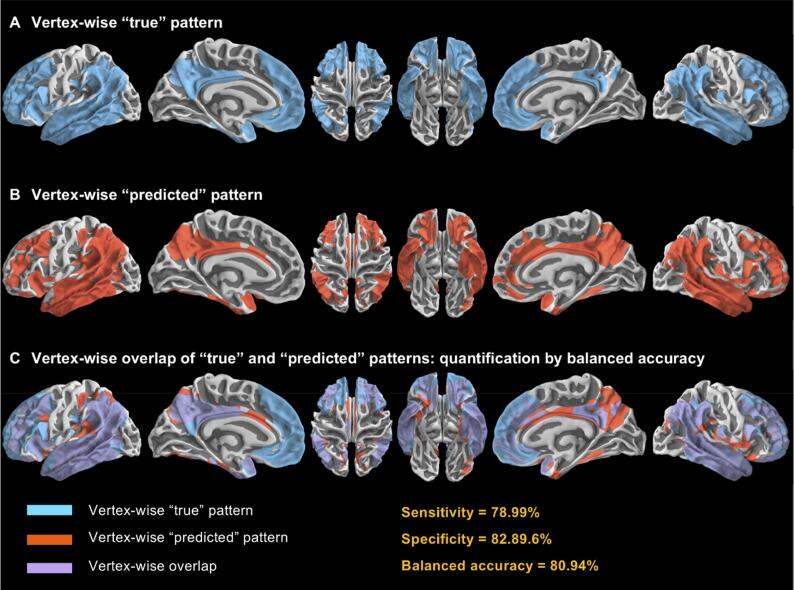
Principle of vertex-wise pattern overlap of “true” and “predicted” patterns. **a** Vertex-wise “true” pattern. **b** Vertex-wise “predicted” pattern. **c** Vertex-wise overlap of “true” and “predicted” patterns quantified by balanced accuracy, which is the average of sensitivity and specificity.

Within the optimal early time frame of eAV45 and among reference regions used for scaling, we thereafter compared the vertex-wise neurodegenerative patterns obtained when comparing i) high-Aβ Alz-CS patients to low-Aβ eHC or ii) high-Aβ AD-d patients to low-Aβ eHC, with eAV45 versus [^18^F]FDG PET smoothed normalized maps (Fig. 1N). Similarly, we compared the patterns of correlations with MMSE and Total Recall scores obtained with eAV45 versus [^18^F]FDG PET smoothed normalized maps across eHC and Alz-CS participants for the optimal early time frame of eAV45 and among reference regions used for scaling (Fig. 1N). To determine these vertex-wise significant patterns we computed threshold-free cluster enhancement statistics (combining the spatial extent of signals) (Smith and Nichols, 2009) from the PALM pipeline with sex, age and education as nuisance covariates. This non-parametric, permutation-based approach for statistical thresholding provides cluster-based inference without the need to specify an arbitrary cluster-forming threshold (as required when applying Gaussian random field theory) (Friston et al., 1996). The number of permutations was set to 10,000, then familywise error (FWE) rate correction was used to correct for multiple comparisons (Holmes et al., 1996), and significant clusters were reported for corrected p-values below 0.05. The vertex-wise overlap between optimal eAV45 and [^18^F]FDG PET significant neurodegenerative patterns was computed as the balanced accuracy (BACC) between “true pattern” ([^18^F]FDG PET vertices: 1 if significant else 0) and “predicted pattern” (optimal eAV45 vertices: 1 if significant else 0), which is the mean between sensitivity and specificity (Fig. 2). In order to robustly quantify the overlap of patterns between optimal eAV45 and [^18^F]FDG PET, we considered 91 statistical thresholds evenly distributed from p = 0.05 to p = 0.001 FWE corrected and then computed BACC associated to each of those statistical thresholds. Finally, to assess the statistical significance of differences among reference regions used for scaling, we performed a non-parametric Friedman test (Hollander et al., 2013) comparing the balanced accuracies of the reference regions used for scaling simultaneously across multiple statistical thresholds, for each of the vertex-wise comparisons or correlations tested.

#### 2.4.4 Machine learning binary classifications between clinical subgroups

Lastly, supervised classification experiments based on linear support vector machine (SVM) algorithm were applied across reference regions successively on optimal eAV45, lAV45, [^18^F]FDG PET, [combined optimal eAV45 and lAV45] and [combined [^18^F]FDG PET and lAV45] measures, derived from mean PET values inside DK parcellation, to compare their discriminative ability for classification of patients with aMCI or AD-d from eHC (Fig. 1O). We used a repeated (N = 250 iterations) hold-out nested cross-validation scheme with class-sizes stratified in the training set (percentage of the smallest class to be reserved for training: 80%) and BACC as metric to measure the performance in order to minimize class-imbalance (Raamana, 2017). Again, we performed a non-parametric Friedman test (Hollander et al., 2013) comparing the ranks of classification performance of the reference regions used for intensity scaling simultaneously across the N iterations of the cross-validation scheme, according to both single (or combined with lAV45) eAV45 vs. [^18^F]FDG PET modalities.

## 3 Results

Main results are presented below based on PVE-non corrected PET data. Results obtained with PVE-corrected PET data for confirmation are summarized at the end of this section.

### 3.1 Vertex-wise within- and inter-subject correlations of eAV45 with [^18^F]FDG PET and lAV45 PET: optimization of the time frame for eAV45

**Fig. 3:**
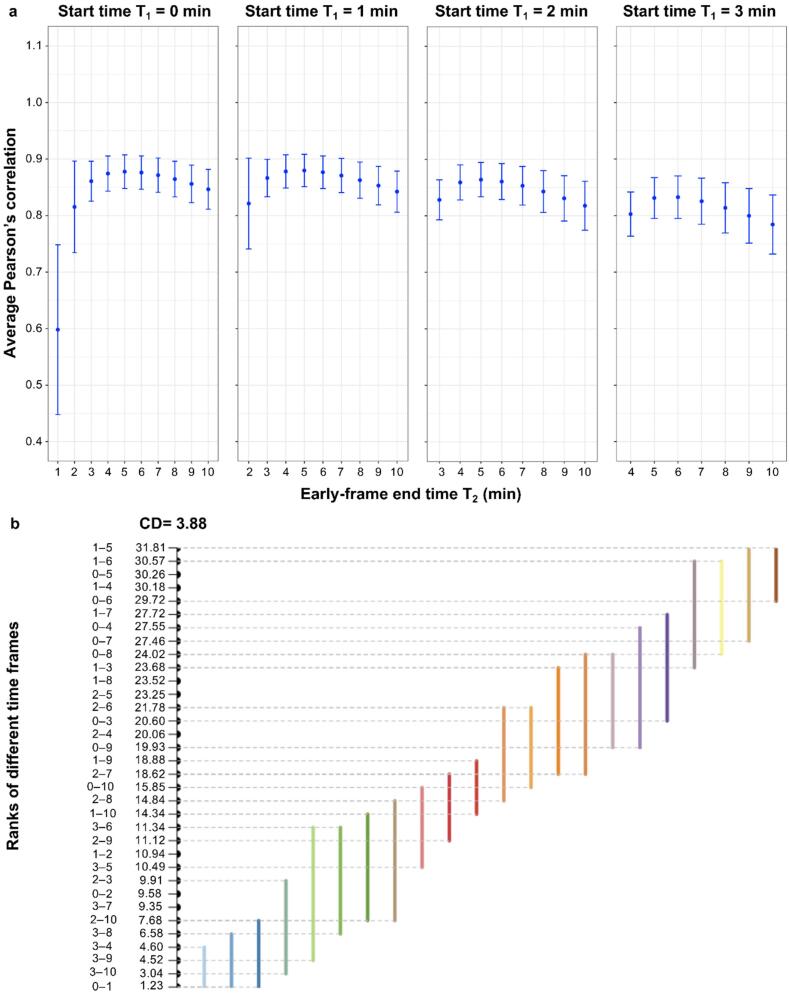
Vertex-wise within-subject correlations of early-phase [^18^F]AV45 PET (eAV45) with [^18^F]FDG PET. **a** Vertex-wise within-subject Pearson’s correlation of smoothed normalized eAV45[T1,T2] with [^18^F]FDG PET maps according to start time T1 (0, 1, 2 or 3 min) and end time T2 (between 1 and 10 min) of early-phase time frames. **b** Critical difference (CD) diagram comparing the ranks of different early-phase time frames in a non-parametric Friedman test based on Pearson’s correlation from a total population of 191 participants. Here, greater numerical values for rank implies higher correlation. Different colored lines here present groups of early-phase time frames that are not significantly different from each other in ranks, each one using a different early-phase time frame as its reference point.

For each starting time T_1_, the vertex-wise within-subject correlations of eAV45 with [^18^F]FDG PET were overall high (most of values between 0.8 and 0.9) and relatively stable, with a trend of increasing rapidly after tracer injection and then decreasing slightly up to T_2_ = 10 min (Fig. 3a). The results from the Friedman and *post-hoc* Nemenyi tests are visualized in a convenient critical difference (CD) diagram as shown in Fig. 3b. The 8 highest ranks (i.e. highest vertex-wise within-subject correlations), that were not statistically significantly different from each other, were connected by a light brown line from the highest ranked early time frame 1–5 min (ranked 31.81) to the lowest ranked early time frame 0–7 min (ranked 27.46).

**Supplementary figure 1:**
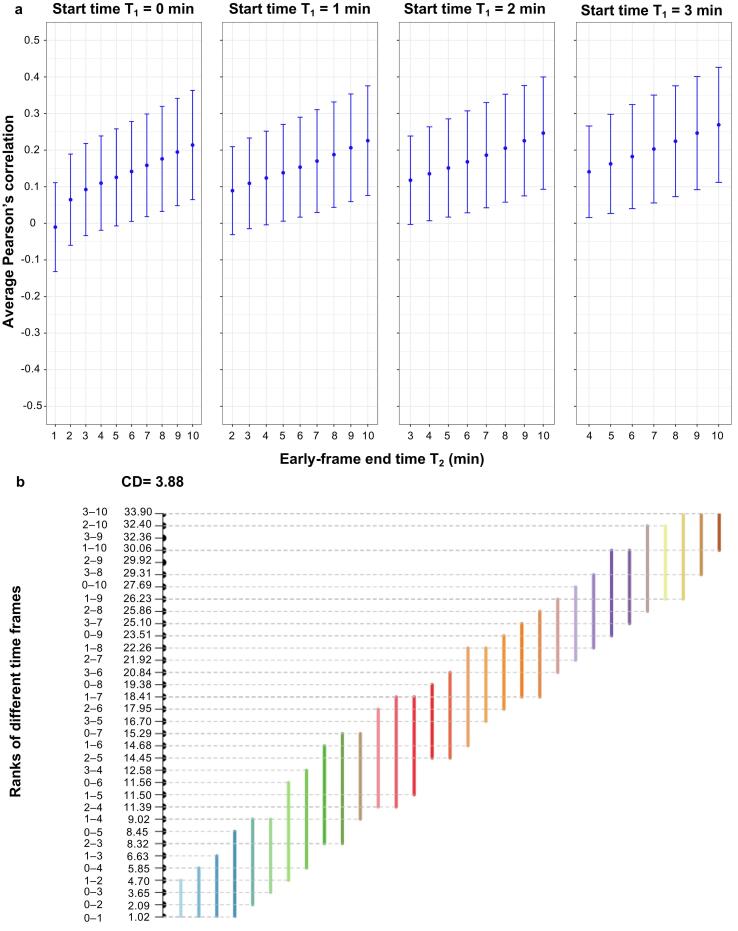


As regard to the vertex-wise within-subject correlations of eAV45 with lAV45, for each starting time T_1_, they linearly increased from tracer injection up to T_2_ = 10 min (Online Supplementary Fig. 1a). The lowest ranks (i.e. lowest vertex-wise within-subject correlations), that were not statistically significantly different from each other, were connected by a blue line from the lowest ranked early time frame 0–1 min (ranked 1.02) to the highest ranked early time frame 0–5 min (ranked 8.45) (Online Supplementary Fig. 1b). Among these lowest vertex-wise within-subject correlations of eAV45 with lAV45, only early time frames 0–4 min (ranked 5.85) and 0–5 min (ranked 8.45) also belonged to the highest vertex-wise within-subject correlations of eAV45 with [^18^F]FDG PET (Fig. 3b).

Based on the significantly 8 highest ranks of within-subject correlations of eAV45 with [^18^F]FDG PET, we performed vertex-wise inter-subject correlations of eAV45 with [^18^F]FDG PET, and eAV45 with lAV45 to precisely determine the best early time frame of eAV45.

**Fig. 4:**
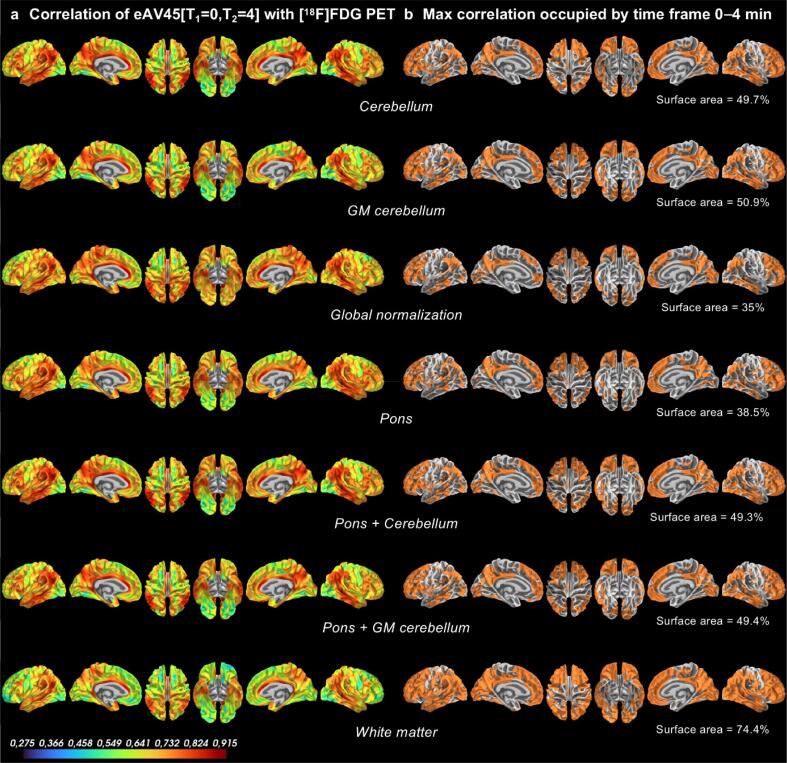
Vertex-wise inter-subject correlations of early-phase [^18^F]AV45 PET (eAV45) with [^18^F]FDG PET. **a** Vertex-wise inter-subject Pearson’s correlation of smoothed normalized eAV45 [T1 = 0,T2 = 4] with [^18^F]FDG PET maps according to the reference region used for intensity scaling. **b** Cortical surface area with maximum inter-subject correlations of eAV45 with [^18^F]FDG PET, occupied by time frame 0–4 min, among the significant highest within-subject correlated time frames and according to the reference region used for intensity scaling.

**Supplementary figure 2:**
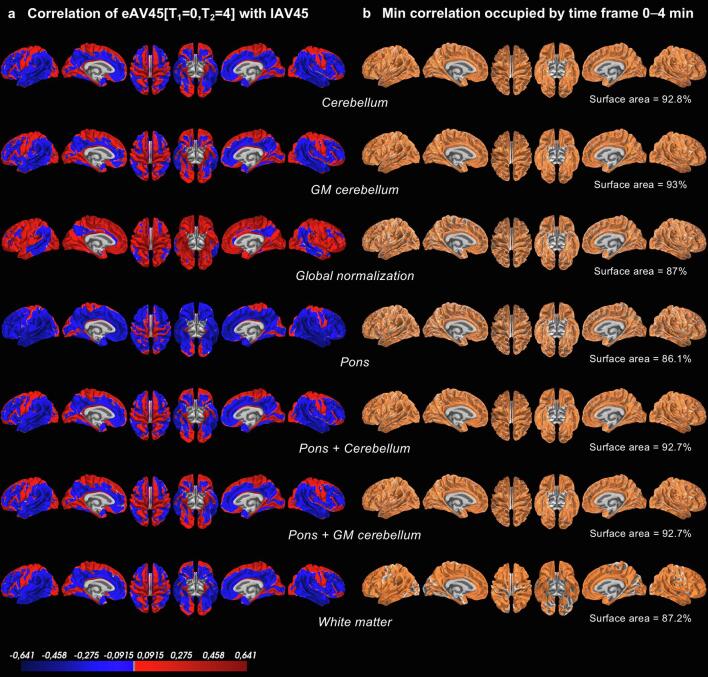


Among the top ranked early time frames of eAV45 and across all reference regions assessed for scaling, vertex-wise inter-subject correlations’ maps of eAV45 with [^18^F]FDG PET consistently presented the same pattern, with high correlation in highly vulnerable regions in AD (medial temporal, lateral temporal and parietal, and superior frontal cortices, temporo-parietal junctions, precuneus, cingulate gyri) (see particular case of early time frame 0–4 min in Fig. 4a). Early time frame 0–4 min presented the maximal cortical surface area (ranging from 35% to 74.4%) with highest inter-subject correlation of eAV45 with [^18^F]FDG PET, whatever the reference region used for scaling (Fig. 4b) compared to other top ranked early time frames (Online Supplementary Table 1). Moreover, early time frame 0–4 min presented the maximal cortical surface area (ranging from 86.1% to 93%) with lowest inter-subject correlation of eAV45 with lAV45, whatever the reference regions used for scaling (Online Supplementary Fig. 2b) compared to the other top ranked early time frames (Online Supplementary Table 2).

Based on vertex-wise within- and inter-subject correlation results of both eAV45 with [^18^F]FDG PET and eAV45 with lAV45, the early time frame 0–4 min of eAV45 appeared to be optimal, among the 34 initially generated early time frames of eAV45, whatever the reference region used for scaling. Following optimization of eAV45 reference region used for scaling was only based on this optimal early time frame.

### 3.2 Clinical subgroups’ comparisons and correlations with neuropsychological scores

**Fig. 5:**
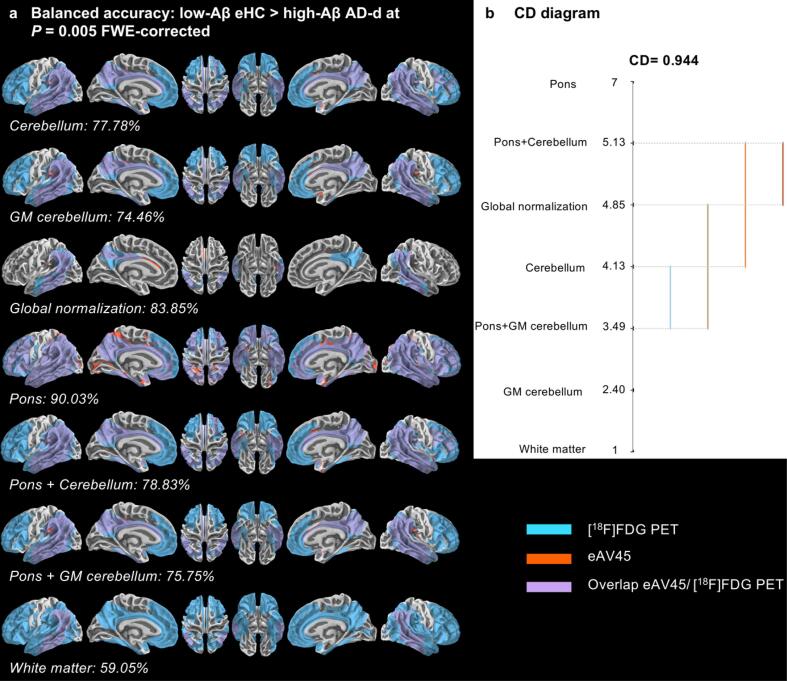
Vertex-wise comparison of patterns of early-phase [^18^F]AV45 PET (eAV45) with [^18^F]FDG PET for the [low-Aβ elderly healthy controls (eHC) > high-Aβ dementia due to AD (AD-d)] contrast. **a** Vertex-wise patterns overlaps, quantified via balanced accuracy, between [^18^F]FDG PET and eAV45 for the [low-Aβ eHC > high-Aβ AD-d] contrast, according to the reference region used for intensity scaling at P = 0.005 family-wise error (FWE)-corrected. **b** Critical difference (CD) diagram comparing the ranks of different reference regions used for intensity scaling in a non-parametric Friedman test based on balanced accuracy at 91p-values evenly distributed from P = 0.05 to P = 0.001 family-wise error (FWE)-corrected.

Whatever the reference region used for scaling, AD-d presented significant vertex-wise hypometabolic patterns in highly vulnerable regions of AD compared to eHC, although less extensive in the particular case of global normalization (only including parietal and lateral temporal cortices) (Fig. 5a).

**Fig. 6:**
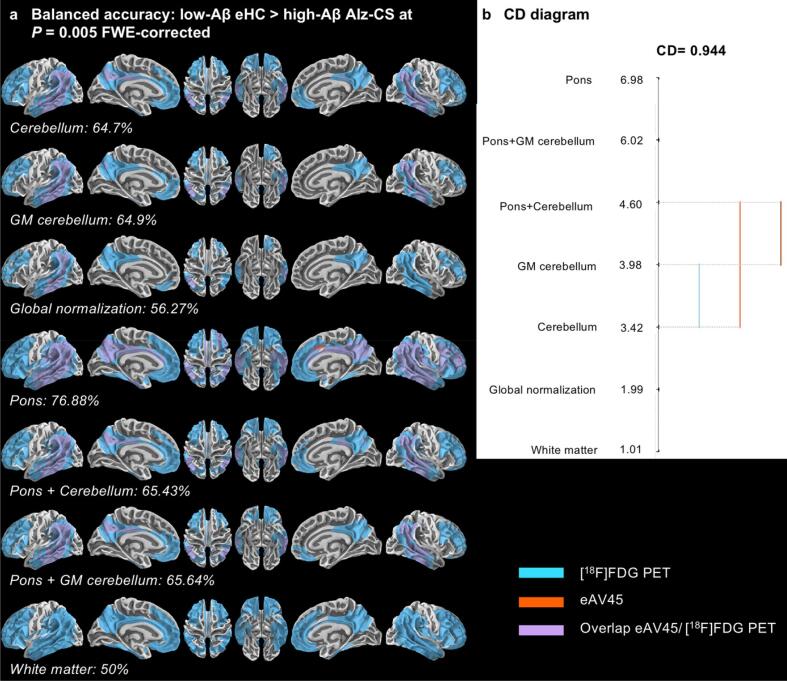
Vertex-wise comparison of patterns of early-phase [^18^F]AV45 PET (eAV45) with [^18^F]FDG PET for the [low-Aβ elderly healthy controls (eHC) > high-Aβ Alzheimer’s clinical syndrome (Alz-CS)] contrast. **a** Vertex-wise patterns overlaps, quantified via balanced accuracy, between [^18^F]FDG PET and eAV45 for the [low-Aβ eHC > high-Aβ Alz-CS] contrast, according to the reference region used for intensity scaling at P = 0.005 family-wise error (FWE)-corrected. **b** Critical difference (CD) diagram comparing the ranks of different reference regions used for intensity scaling in a non-parametric Friedman test based on balanced accuracy at 91p-values evenly distributed from P = 0.05 to P = 0.001 family-wise error (FWE)-corrected.

Optimal early time frame of eAV45 in AD-d presented also significant hypoperfusion patterns compared to eHC in highly vulnerable regions of AD but in a less sensitive manner than [^18^F]FDG PET (Fig. 5a), with best vertex-wise overlap (i.e. BACC) between eAV45 and [^18^F]FDG PET in the case of pons scaling (Fig. 5a). The results from the Friedman and *post-hoc* Nemenyi tests are visualized in a convenient CD diagram as shown in Fig. 5b. Among the 91 evenly distributed statistical thresholds, Friedman test confirmed that pons scaling had statistically significant best BACC (ranked 7) compared to other reference regions. Similar results were found when comparing Alz-CS to eHC with pons scaling having the best BACC (ranked 6.98) (Fig. 6).

**Fig. 7:**
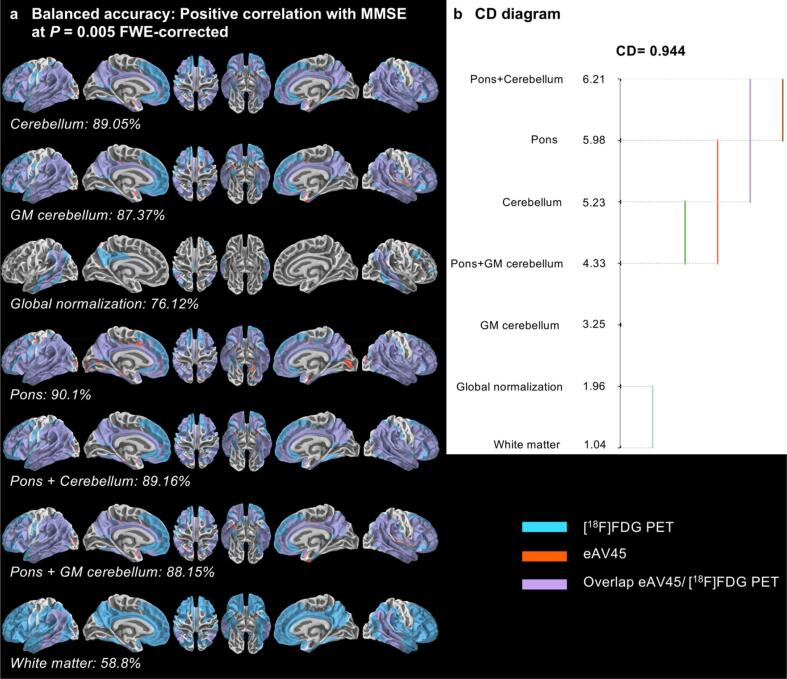
Vertex-wise comparison of patterns of early-phase [^18^F]AV45 PET (eAV45) with [^18^F]FDG PET for the positive correlation with mini mental state examination (MMSE). **a** Vertex-wise patterns overlaps, quantified via balanced accuracy, between [^18^F]FDG PET and eAV45 for positive correlation with MMSE, according to the reference region used for intensity scaling at P = 0.005 family-wise error (FWE)-corrected. **b** Critical difference (CD) diagram comparing the ranks of different reference regions used for intensity scaling in a non-parametric Friedman test based on balanced accuracy at 91p-values evenly distributed from P = 0.05 to P = 0.001 family-wise error (FWE)-corrected.

Vertex-wise positive correlations of [^18^F]FDG PET with MMSE showed widespread patterns of correlation whatever the reference region used, although less extensive in the particular case of global normalization (Fig. 7a). Optimal early time frame of eAV45 presented also significant vertex-wise positive correlation with MMSE, but in a less sensitive manner than [^18^F]FDG PET particularly in the case of WM scaling (Fig. 7a). Statistically significant best BACC were found for combined cerebellum and pons (ranked 6.21), pons (ranked 5.98) and cerebellum (ranked 5.23) compared to other reference regions (Fig. 7b).

**Supplementary figure 3:**
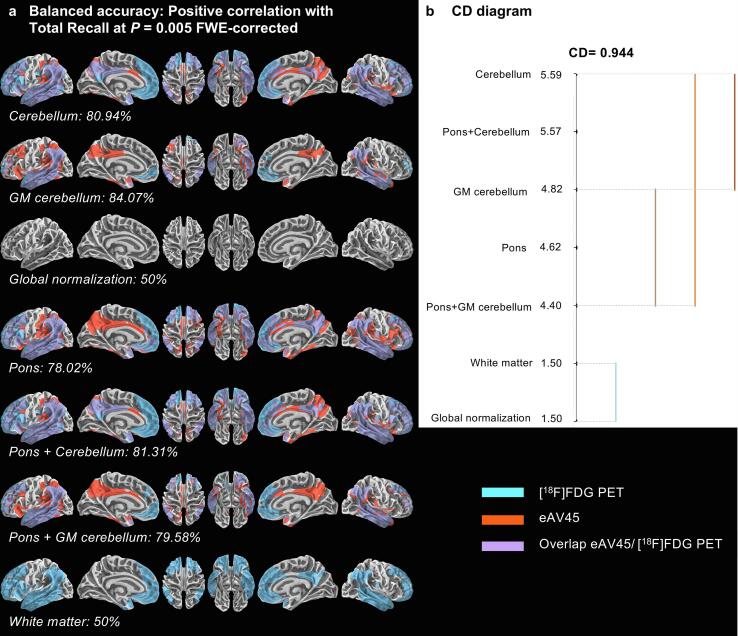


Finally, most of the reference regions, besides WM scaling and global normalization, showed good and statistically similar vertex-wise overlaps between eAV45 and [^18^F]FDG PET (ranked from 4.4 to 5.59, Online Supplementary Fig. 3b), regarding positive correlation with Total Recall in regions linked to memory (temporal lobes, orbito-frontal, lateral and medial parietal cortices) (Online Supplementary Fig. 3a). Among all these vertex-wise clinical subgroups’ comparisons and correlations with neuropsychological scores, both global normalization and WM scaling performed worse in terms of BACC.

Based on quantified overlaps between eAV45 and [^18^F]FDG PET resulting from vertex-wise clinical subgroups’ comparisons and correlations with neuropsychological scores, while pons normalization tended to give the best results, both global normalization and white matter reference regions appeared to give the worst results. Following optimization of eAV45 reference region used for scaling consequently excluded both global normalization and white matter reference regions.

**Supplementary figure 4:**
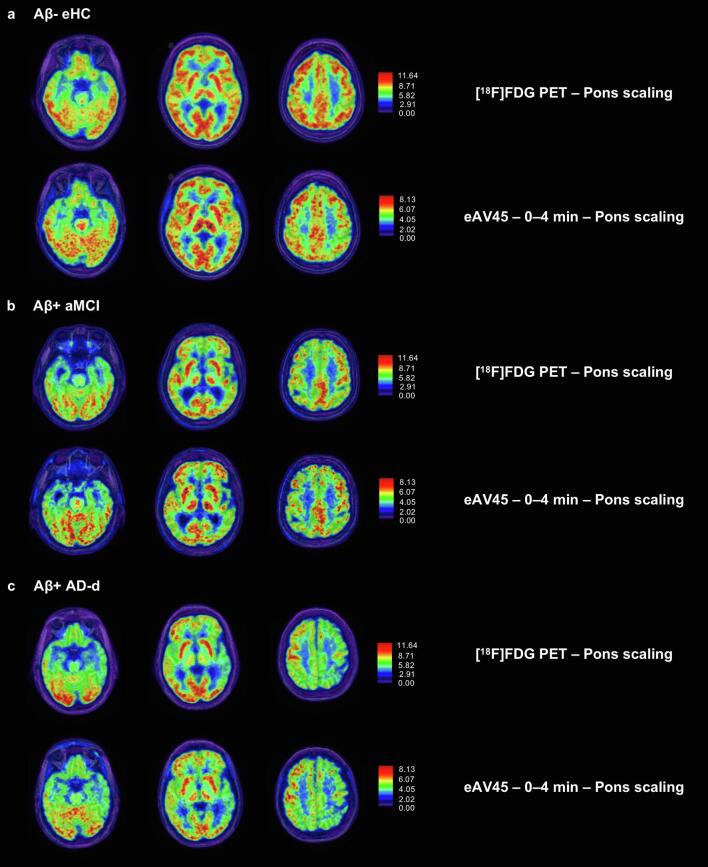


The quantitative similarity presented above between both [^18^F]FDG PET and eAV45 can be also observed qualitatively on individual scans, with similar AD specific patterns in both the Aβ + aMCI and the AD-d individuals (see online Supplementary Fig. 4 for the sake of illustration).

**Supplementary figure 5:**
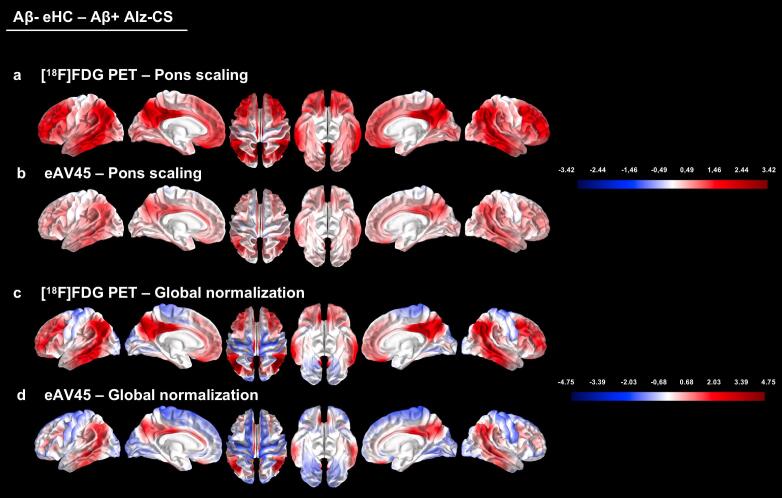


When exploring the difference between means of smoothed Aβ- eHC individuals’ images and smoothed Aβ + Alz-CS individuals’ images for both [^18^F]FDG PET and eAV45 (Online Supplementary Fig. 5), a clear difference was observed between pons scaling and global normalization. When pons scaling was used the difference image for eAV45 seemed to be close but at lower sensitivity from the difference image for [^18^F]FDG PET, while when global normalization was used the difference image for eAV45 seemed to have divergent patterns, notably in frontal and orbito-frontal regions, compared to those of the difference image for [^18^F]FDG PET.

### 3.3 Machine learning binary classifications: performance comparison between eAV45 and [^18^F]FDG PET

Results from binary classifications of pairs of clinical groups according to single or combined modalities were only presented for the most promising pons scaling, although similar results were obtained for the four other reference regions.

**Fig. 8:**
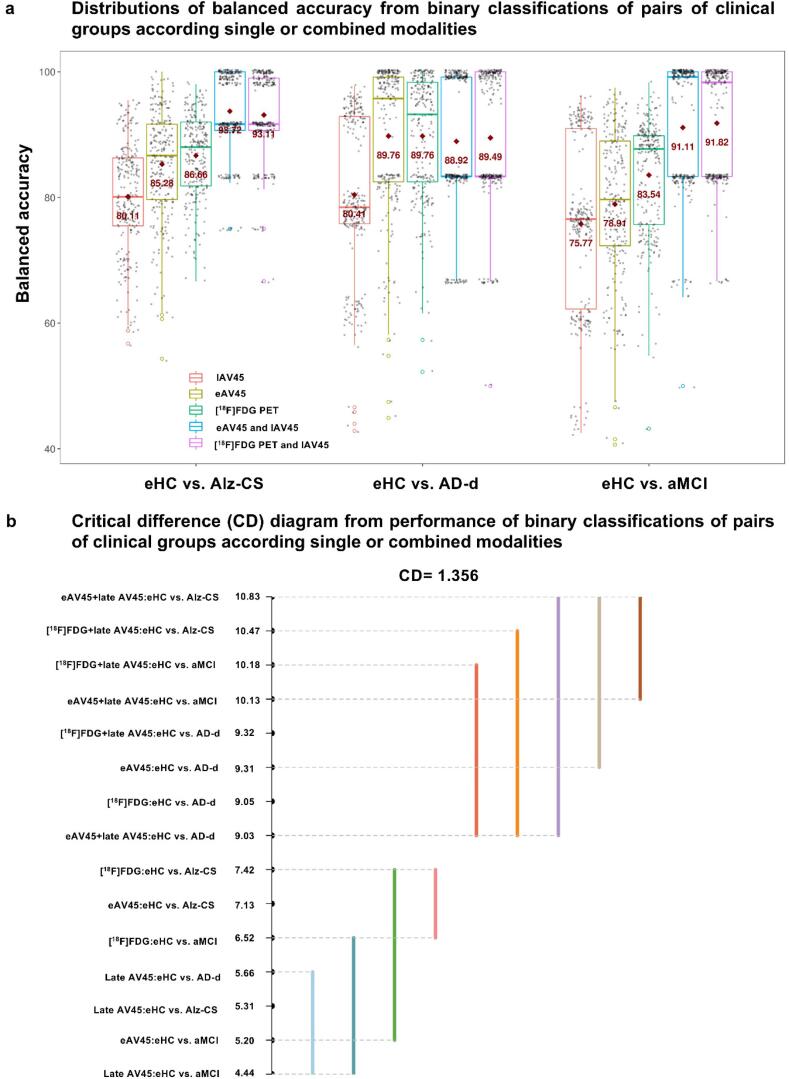
Binary classification performance of pairs of clinical groups according to single or combined modalities. **a** Distributions of balanced accuracy for each binary classification (eHC vs. Alz-CS, eHC vs. AD-d or eHC vs. aMCI) according to each single (early-phase [18F]AV45 PET (eAV45), late-phase [18F]AV45 PET (lAV45) or 18F]FDG PET) or combined ([eAV45 and lAV45] or [[18F]FDG PET and lAV45]) modalities. The performance presented here is a distribution of balanced accuracy values from repeated (N = 250 iterations) hold-out nested cross-validation (CV) scheme with class-sizes stratified in the training set (whose mean is shown with a red cross-hair symbol). **b** Critical difference (CD) diagram comparing the ranks of classification performance for each binary classification (eHC vs. Alz-CS, eHC vs. AD-d or eHC vs. aMCI) according to each single (early-phase [18F]AV45 PET (eAV45), late-phase [18F]AV45 PET (lAV45) or 18F]FDG PET) or combined ([eAV45 and lAV45] or [[18F]FDG PET and lAV45]) modalities. Here, greater numerical values for rank implies higher classification performance. Different colored lines here present groups of [biomarker:binary classification] pair that are not significantly different from each other in ranks, each one using a different [biomarker: binary classification] pair as its reference point.

Performances of eAV45 vs. [^18^F]FDG PET in the binary classifications of eHC vs. Alz-CS, eHC vs. AD-d and eHC vs. aMCI (Fig. 8a), were not significantly different from each other (ranked 7.13 vs. 7.42, 9.31 vs. 9.05, and 5.20 vs. 6.52, respectively, Fig. 8b). eAV45 gave better performances than lAV45 significantly in the binary classification of eHC vs. AD-d (ranked 9.31 vs. 5.66), while non-significantly in the binary classifications of eHC vs. Alz-CS and eHC vs. aMCI (ranked 7.13 vs. 5.31, and 5.20 vs. 4.44, respectively, Fig. 8).

Combined eAV45 and lAV45 always gave significantly better performances in the binary classifications of eHC vs. Alz-CS and eHC vs. aMCI than eAV45 alone (ranked 10.83 vs. 7.13, and 10.13 vs. 5.20, respectively), [^18^F]FDG PET alone (ranked 10.83 vs. 7.42, and 10.13 vs. 6.52, respectively) or lAV45 alone (ranked 10.83 vs. 5.31, and 10.13 vs. 4.44, respectively, Fig. 8). For eHC vs. AD-d binary classification, combined eAV45 and lAV45 gave better performance than lAV45 alone (ranked 9.03 vs. 5.66), while giving similar performances than eAV45 alone (ranked 9.03 vs. 9.31) or [^18^F]FDG PET alone (ranked 9.03 vs. 9.05, Fig. 8).

Finally, performances of combined eAV45 and lAV45 vs. combined [^18^F]FDG PET and lAV45 in the binary classifications of eHC vs. Alz-CS, eHC vs. AD-d and eHC vs. aMCI were not significantly different from each other (ranked 10.83 vs. 10.47, 9.03 vs. 9.32, and 10.13 vs. 10.18, respectively, Fig. 8).

### 3.4 Brief description of the results based on PVE-corrected PET data

Results were in accordance with those obtained from the PVE-non corrected PET data, despite (i) [^18^F]FDG PET had slightly non-significant better performance in the binary classifications of eHC vs. Alz-CS and eHC vs. aMCI than eAV45; (ii) eAV45 and lAV45 had similar performance in the binary classification of eHC vs. Alz-CS; and finally (iii) combined eAV45 and lAV45 had slightly non-significant better performance in the binary classification of eHC vs. AD-d than [^18^F]FDG PET (data not shown).

## 4 Discussion

The present study showed that the early time frame 0–4 min was optimal by maximizing both within- and inter-subject correlations of eAV45 with [^18^F]FDG PET, while minimizing both within- and inter-subject correlations of eAV45 with lAV45. Balanced accuracies of pattern overlap, derived from associations of both [^18^F]FDG PET and eAV45 with clinical diagnosis and cognition, were globally maximal with pons scaling, whereas classification performance between patients’ clinical subgroups and healthy controls were similar across reference regions for both [^18^F]FDG PET and eAV45. Finally, classification performance was significantly superior for combined eAV45 and lAV45 compared to [^18^F]FDG PET alone, eAV45 alone or lAV45 alone, and similar to combined [^18^F]FDG PET and lAV45.

Optimization and validation of eAV45 is a critical and timely issue (Valentina et al., 2016). Our dataset is optimal for addressing this issue with a large number of dual-phase [^18^F]AV45 PET exams acquired on the same scanner in a population ranging from healthy young to elderly volunteers with no cognitive impairment to Alzheimer’s clinical syndrome with MCI or dementia. Validating the results on a mixed study population provides further support that optimal early time frame and preprocessing methods of eAV45 are not dependent on clinical diagnosis, suggesting wider applicability of our methodology. While previous studies (Asghar et al., 2018, Hsiao et al., 2012, Kuo et al., 2017, Lin et al., 2016, Ottoy et al., 2019) chose the optimal early time frame of eAV45 based solely on the best within- or inter-subject correlation with [^18^F]FDG PET SUVR values, this study considers for the first time many complementary quantitative analyses to robustly optimize both the early time frame and the preprocessing methods of eAV45, by comprehensively assessing the similarities and differences between eAV45 and [^18^F]FDG PET. Moreover, this study contrasted the discriminatory power of eAV45 and [^18^F]FDG PET in individual cases using (i) a robust cross-validation scheme to avoid overly optimistic classification performance and (ii) a whole-brain data-driven approach rather than specific ROIs to better compare performance between both modalities. Finally, the use of cortical surface-based analysis in PET have resulted in substantial improvements in the reliability and detectability of effects (Greve et al., 2014).

Within-subject correlations of eAV45 and [^18^F]FDG PET distributions were globally high and similar to published studies (Asghar et al., 2018, Fu et al., 2014, Hsiao et al., 2012, Joseph-Mathurin et al., 2018, Ottoy et al., 2019, Rodriguez-Vieitez et al., 2016, Rostomian et al., 2011), and generally associated with middle-to-high vertex-wise inter-subject correlations between both modalities irrespective of the reference region. Higher correlations in vulnerable regions in AD may be explained by a greater dynamic range of metabolism and perfusion within these regions due to our mixed study population ranging from healthy controls to Alzheimer’s clinical syndrome with MCI or dementia. Our results suggest that early time frame of 0–4 min is optimal by maximizing vertex-wise both within- and inter-subject correlations of eAV45 with [^18^F]FDG PET, while minimizing vertex-wise both within- and inter-subject correlations of eAV45 with lAV45, whatever the reference region used for scaling and independently of the PVE. This optimal early time frame slightly differed from previous studies suggesting either 0–2 min (Ottoy et al., 2019) or 1–6 min (Hsiao et al., 2012) as the optimal early time frame of eAV45 with the best association with [^18^F]FDG PET. Discrepancies with previous studies may arise from multiple factors including: (i) our sample size of 191 participants compared to relatively small sample sizes of 39 (Ottoy et al., 2019) and 7 (Hsiao et al., 2012) participants previously; (ii) the use of complementary quantitative analyses (within- and inter-subject correlations) to optimize the early time frame of eAV45, contrary to within-subject correlations only for previous studies; (iii) the minimization of the correlations of eAV45 with lAV45 to avoid early Aβ binding contamination within eAV45 signal, not previously considered; (iv) the validation of the optimal early time frame of eAV45 on both non-PVC and PVC results, contrary to only PVC (Ottoy et al., 2019) or non-PVC (Hsiao et al., 2012) results previously; (v) the validation of the optimal early time frame of eAV45 on multiple reference regions, contrary to only one or two reference regions previously; and finally (vi) our vertex-wise finer scale of analysis, contrary to region-based (Ottoy et al., 2019) or voxel-wise (Hsiao et al., 2012) scales previously. Moreover, our optimal time frame 0–4 min of eAV45 starts at the time of injection, ensuring the record from the initial phase of tracer influx up to the time of peak concentration which occurred within 4 min of tracer administration. Lastly, a previous work showed that restricting the early time frame 0–2 min of eAV45 instead of 1–6 min provides more CBF-like than [^18^F]FDG-like information (Hsiao et al., 2012).

[^18^F]FDG PET showed significant cortical glucose metabolism decreases in Alzheimer’s clinical syndrome participants compared to eHC and the extent of these decreases were wider with disease severity. Moreover, these decreases were well associated with global cognitive and episodic memory impairments. eAV45 with pons scaling showed significant cortical hypoperfusion patterns best overlapping with hypometabolic patterns of [^18^F]FDG PET, compared to other reference regions. However, whatever the reference region used for scaling, the extent of hypometabolic patterns was generally higher than the extent of hypoperfusion patterns, particularly in the prodromal AD stage. The frontal cortex for instance is altered using [^18^F]FDG PET; with eAV45, it is not detected at the same threshold but only when using a more lenient threshold (data not shown). This is in agreement with previous studies showing that changes in early amyloid PET distribution between subject group seemed to reasonably well approximate those of [^18^F]FDG PET, but at the cost of lower sensitivity (Forsberg et al., 2012, Fu et al., 2014, Hsiao et al., 2012, Ottoy et al., 2019, Segovia et al., 2018). This might reflect the fact that eAV45 only measure blood perfusion deficits while [^18^F]FDG PET is sensitive to additional processes leading to glucose consumption default above hypoperfusion. Moreover, the observation of relatively preserved perfusion in metabolically deficient regions (such as frontal cortices) or relatively preserved metabolism in perfusion deficient regions (such as medial parietal cortices) could be explained by a regional neurovascular decoupling in the resting brain (Gur et al., 2008). Finally, our knowledge is still incomplete regarding the spatiotemporal relationships between brain perfusion and metabolism, which may not necessarily follow a consecutive progression (Besson et al., 2015).

Regarding reference regions, the pons appeared as the best choice in terms of quantified overlaps between eAV45 and [^18^F]FDG PET when evaluating cross-sectional associations with disease severity and cognition, above the cerebellum, while both global normalization and WM reference regions generally were the worst. The pons has been shown to be a more stable reference region than the cerebellum (Shokouhi et al., 2016a, Shokouhi et al., 2016b), since cerebellar perfusion can itself be affected by cross cerebellar diaschisis in AD which might propagate to bias in normalized SUV calculations. In addition, previous studies have shown that the cerebellum is relatively hyperperfused compared to its rate of glucose metabolism (Gur et al., 2008, Hsiao et al., 2012), suggesting that cerebellum may not be considered as the best reference region for screening perfusion changes in AD. The lower performance of the global normalization procedure was expected given that the global measure used for scaling is influenced by the effect of the pathology. Previous studies have shown that the cerebral WM appears as the optimal reference region for longitudinal lAV45 studies where values are more stable (Fleisher et al., 2017). However, we showed here that it is not the case for cross-sectional eAV45 measurements. It is possible that the signal in the WM is also influenced by the pathology or that the signal in the WM is less reliable to measure inter-individual variability of eAV45 measurements.

eAV45 showed globally similar classification performance for eHC vs. Alz-CS, eHC vs. AD-d, and eHC vs. aMCI to that of [^18^F]FDG PET whatever the reference region used. Furthermore, the combination of eAV45 and lAV45 significantly improved the classification performance for distinguishing both Alz-CS from eHC and aMCI from eHC compared to [^18^F]FDG PET alone or lAV45 alone, whatever the reference region used. This suggests that, for an equal number of tracer injection, dual-phase [^18^F]AV45 PET outperformed the classification performance of [^18^F]FDG PET and lAV45. Thus, the combination of [^18^F]AV45 PET dual-biomarker for aMCI classification is necessary and valuable (Fu et al., 2014, Li et al., 2008). Finally, contrary to our results showing similar classification performance for the combination of [^18^F]AV45 PET dual-biomarker and the combination of [^18^F]FDG PET and lAV45, Fu et al. (2014) showed that the combination of [^18^F]FDG PET and lAV45 had better performance, for the classification of eHC vs. aMCI, than the combination of [^18^F]AV45 PET dual-biomarker. This may be explained (i) by the use of only 4 composite ROIs derived from AAL atlas rather than a whole-brain data-driven approach to compare performance between both modalities, and (ii) by the use of leave-one-out cross-validation rather than repeated hold-out nested cross-validation scheme, what could provide overly optimistic classification performance.

This study could facilitate biomarker-based research in allowing to get Aβ and neurodegeneration highly complementary information from a single [^18^F]AV45 PET scan. Therefore, characterization of neuronal activity, a proxy for neurodegeneration, alongside amyloidosis, is key to a more complete understanding of cognitive decline. It would also have clinical implications since the combination of these complementary Aβ and neurodegeneration information from a single [^18^F]AV45 PET scan would improve the diagnosis, by providing simultaneous information on the underlying pathology (Aβ) and on the disease stage with predictive power of short-term outcome (neurodegeneration). Moreover, for participants already receiving an [^18^F]AV45 PET scan for assessment of Aβ deposition, substitution of eAV45 for [^18^F]FDG PET would minimize costs, examination time, radiation exposure and thus participant burden by acting as a surrogate for the [^18^F]FDG PET scan. There is still a need to validate the use of eAV45 as an alternative of [^18^F]FDG PET in longitudinal studies (e.g., to monitor the progression of AD or assessing a treatment response in a clinical trial), since potential differences between eAV45 and [^18^F]FDG PET might be of importance when small effect size are relevant (see also above). Furthermore, the question of the optimal reference region for eAV45 in longitudinal assessment should be specifically assessed, which could lead to a conclusion differing from this cross-sectional study – as it was the case for lAV45. Future studies should evaluate whether eAV45 proxy can replace [^18^F]FDG PET on a single subject level for clinical purposes in the differential diagnosis of dementia. In addition, due to the mechanistic similarity between AV45 and the other commercially available amyloid imaging agents ([^11^C]PiB, [^18^F]Florbetaben and [^18^F]Flutemetamol), there would be widespread clinical use potential in translating our comprehensive methodology to one or more of these agents or even to possibly one of the tau imaging agents as described previously (Rodriguez-Vieitez et al., 2017). Finally, a medium-term development would be the integration into PET-scanner of a readily available software to optimally extract the [^18^F]AV45 PET dual information.

Previous studies showed that the relative delivery rate R_1_, derived from pharmacokinetic modeling or simplified reference tissue model (SRTM) of the dynamic amyloid-PET scan as the ratio of the first-pass influx rate (K1) to its reference region value, could be a closer proxy of perfusion and synaptic function than early amyloid PET (Bilgel et al., 2019, Joseph-Mathurin et al., 2018, Ottoy et al., 2019). However, R_1_ images has the disadvantage of requiring a long dynamic scanning protocol (subject to patient motion and discomfort) (Shokouhi et al., 2016a, Shokouhi et al., 2016b) and are generally noisy, forcing the need of further processing including noise reduction for clinical application (Hsiao et al., 2012). Thus, R_1_ would only be advised in clinical research setting in which high accuracy is needed. Alternatives would nevertheless be possible as simultaneous ASL MRI and amyloid PET or pharmacokinetic modeling of non-invasive dual-time window acquisition (Bullich et al., 2018). Another limitation is that cerebellum and subcortical structures have not be assessed in this cortical surface-based study. While it could be interesting to verify in these regions the consistency of results compared to cortical areas, the similarities of eAV45 with [^18^F]FDG PET would be biased in these regions since there are known to be hyperperfused with respect to their glucose metabolism (Gur et al., 2008, Hsiao et al., 2012). In addition, differences of eAV45 uptake in very localized subcortical regions would not influence the image content and thus the diagnostic accuracy.

## 5 Conclusion

In conclusion, this study shows that eAV45 from 0 to 4 min with pons scaling is an optimal surrogate of [^18^F]FDG PET in ageing and Alzheimer’s clinical syndrome. The strong potential of optimized dual-phase [^18^F]AV45 PET is highlighted by the capacity to outperform at the individual level the discriminative power of [^18^F]FDG PET or lAV45 alone, when combining both eAV45 and lAV45 information obtained from a single PET-tracer injection. Interestingly, the use of dual-phase [^18^F]AV45 PET instead of [^18^F]FDG PET plus lAV45 will reduce the radiation dose, total time and number of visits and costs.

## 6 Data statement

The datasets used for the present work, with the exception of the participant PET and MRI images, are available on request from the corresponding author pending the institute Ethics approval.

## Footnotes

1 http://dbm.neuro.uni-jena.de/cat/
