# Supplementary material for: Evaluation of the early-phase [18F]AV45 PET as an optimal surrogate of [18F]FDG PET in ageing and Alzheimer’s clinical syndrome
Source: Neuroimage Clin. 2021 Jul 1;31:102750. doi: 10.1016/j.nicl.2021.102750 (PMC8274342; doi:10.1016/j.nicl.2021.102750)
Supplement: Supplementary data 1 [file mmc1.docx]

| Online Supplementary Table 1 | | | | | | | | |
| --- | --- | --- | --- | --- | --- | --- | --- | --- |
| Cortical surface area of maximum correlation of eAV45 with [^18^F]FDG PET | | | | | | | | |
|  | 0−4 min | 0−5 min | 0−6 min | 0−7 min | 1−3 min | 1−4 min | 1−5 min | 1−6 min |
| Reference region for scaling |  |  |  |  |  |  |  |  |
| Cerebellum | 49.7% | 16.5% | 7.7% | 6.5% | 10.2% | 4.7% | 2.7% | 2.1% |
| GM cerebellum | 50.9% | 14.7% | 7.3% | 5.7% | 11.3% | 5% | 2.9% | 2.2% |
| Global normalization | 35% | 16.5% | 9.3% | 10.8% | 8.6% | 6.6% | 5.5% | 7.6% |
| Pons | 38.5% | 23.1% | 13.4% | 7.8% | 7.2% | 5.7% | 2.8% | 1.5% |
| Pons + Cerebellum | 49.3% | 16.7% | 7.9% | 6.7% | 10.1% | 4.6% | 2.7% | 2.1% |
| Pons + GM cerebellum | 49.4% | 15.3% | 7.9% | 6.4% | 10.9% | 4.9% | 3% | 2.3% |
| White matter | 74.4% | 14.2% | 4.3% | 2.4% | 3.1% | 1.1% | 0.3% | 0.2% |
| *eAV45* early-phase [^18^F]AV45 PET | | | | | | | | |

| Online Supplementary Table 2 | | | | | | | | |
| --- | --- | --- | --- | --- | --- | --- | --- | --- |
| Cortical surface area of maximum correlation of eAV45 with lAV45 | | | | | | | | |
|  | 0−4 min | 0−5 min | 0−6 min | 0−7 min | 1−3 min | 1−4 min | 1−5 min | 1−6 min |
| Reference region for scaling |  |  |  |  |  |  |  |  |
| Cerebellum | 92.8% | 1.6% | 0.2% | 0.1% | 4.8% | 0.3% | 0.1% | 0.1% |
| GM cerebellum | 93% | 1.4% | 0.2% | 0.1% | 4.8% | 0.3% | 0.1% | 0.1% |
| Global normalization | 87% | 3% | 0.7% | 0.4% | 7.2% | 1.1% | 0.3% | 0.3% |
| Pons | 86.1% | 3.1% | 0.3% | 0.2% | 9.3% | 0.8% | 0.1% | 0.1% |
| Pons + Cerebellum | 92.7% | 1.7% | 0.2% | 0.1% | 4.9% | 0.3% | 0.1% | 0.1% |
| Pons + GM cerebellum | 92.7% | 1.5% | 0.2% | 0.1% | 5% | 0.3% | 0.1% | 0.1% |
| White matter | 87.2% | 1.4% | 0.2% | 0% | 10.6% | 0.6% | 0% | 0% |
| *eAV45* early-phase [^18^F]AV45 PET, *lAV45* late-phase [^18^F]AV45 PET | | | | | | | | |
